# Regulations on nutrition in Indonesia and its relation to early childhood caries

**DOI:** 10.3389/fpubh.2022.984668

**Published:** 2022-09-28

**Authors:** Rosa Amalia, Fitrina R. Siregar, M. Fahmi Alfian, Leny P. Arie Sandy

**Affiliations:** Faculty of Dentistry, Gadjah Mada University, Yogyakarta, Indonesia

**Keywords:** nutrition, ECC, children, Indonesia, policy

## Abstract

There is a close relationship between food and nutrition policies and oral health. The relationship between nutrition and dental problems has been widely discussed, including the major dental problem in children: early childhood caries (ECC). Health-oriented national policies are the main principles of public health welfare. This article is a policy brief that provide a review of the food and nutrition policies in Indonesia that may have a relationship with ECC. It is concluded that some policies support the efforts to prevent ECC however, other technical explanations are still needed for health workers and especially parents regarding its simple implementation in everyday life. Multisectoral approaches that includes health, nutrition and education are needed to address ECC.

## Introduction

Early childhood caries (ECC) is the most common chronic infectious disease in children aged <71 months. The World Health Organization (WHO) has represented the ECC as a global problem since the prevalence is increasing rapidly in low and middle-income countries and particularly frequent or severe among children living in deprived communities (1). ECC occurs due to the demineralization of teeth caused by acids from dietary sugars metabolized by oral bacterial strains (1). Other risk factors are prolonged bottle feeding, frequent snacking and consumption of sugary drinks, inadequate tooth brushing, and lack fluoride and dental care (2). There was a significant association between ECC and malnutrition; a two-way relationship is postulated; malnutrition affects the occurrence of caries, and caries affects the occurrence of malnutrition in children (3, 4). Based on the 2018 Indonesia's Basic Health Research report, the prevalence of caries in primary teeth in children aged 3–4 is 81.5%, and aged 5 years is 90.2% (5).

Evidence suggests that food and nutrition which play a role in the growth and development of children also have impacts on the occurrence of ECC eventually (2). Thus, this policy brief offers a review of the opportunities in the existence of the food and nutrition policies to prevent and control early childhood caries (ECC) in Indonesia and discusses actions for decisions makers to decrease the prevalence of ECC through effective regulations.

## Sections on policy options and implications

### Existing foods and nutritions policy in Indonesia that potentially has a role in preventing and controlling ECC

Indonesia is the largest archipelagic country in the world and the most populous nation in Southeast Asia (6). Indonesia is a middle-income country with positive trends in economic growth, life expectancy, and increased food security from year to year (7). The Indonesian diet highly depends on one staple food (rice) and low meat and fat consumption, while consumption of food sources of fruit and vegetables is only half of the recommended daily intake and decreases over time (8). The following are six food and nutrition policies in Indonesia which have aim to ensure the growth and development and as well healthy eating of the children and considered have the potential to support ECC prevention efforts.

#### Ministerial Regulation on Health no. 51 of 2016 concerning nutritional supplementation product standards

The Nutritional Supplementation Policy aims to achieve nutritional adequacy for infants, toddlers, school-age children, women of childbearing age, pregnant women, and postpartum mothers as a vulnerable group (9). Nutritional supplementation is the addition of food or nutritional substances that are given in the form of additional food, basic added tablets, vitamin A capsules, and nutritional powder. Every nutritional supplementation product circulating in Indonesia must meet standards, including those regarding content, food additives, microbial and heavy metal contamination, processing, and package labeling. Nutritional fortification for children under the age of 5 contains at least 12 vitamins and four minerals: Vitamins A, B1, B2, B3, B6, B12, C, D3, E, and K1, folate, pantothenic acid, Iodine, iron, zinc, selenium, and maltodextrin.

This Regulation has a potential role in ECC prevention since several nutrients, including calcium, fluorine, phosphorus, vitamin A and vitamin D, have a significant function in the formation of tooth morphology, chemical composition, and tooth eruption pattern (10). A lack of these nutrients since maternal period can affect the resistance of teeth to caries. Increased maternal vitamin D and calcium intake reduce the risk of early childhood caries (10, 11).

#### Ministerial Regulation on Health no. 30 of 2013 concerning inclusion of information on sugar, salt, and fat content and health messages for processed foods and ready-to-eat foods

This policy aims to reduce the risk of non-communicable diseases, such as hypertension, stroke, diabetes, and heart attacks caused by excessive sugar, salt, and fat intake in processed or ready-to-eat foods (12). The sugar, salt, and fat content in question is the total sugar content, both monosaccharides, and disaccharides, total sodium in the form of mineral compounds with the main elements sodium and chloride, and total fat, namely the content of fatty acids expressed as triglycerides. Thus, the health message is that consuming more than 50 grams of sugar, more than 2,000 milligrams of sodium, or more than 67 grams of total fat per person per day places an individual at risk of non-communicable disease and keeps healthy. Concerning ECC, information on sugar content is very important since continuous consumption of high sugary foods and beverages, and lack of adequate fluoride levels can accelerate the occurrence of ECC (13). Consumption of cariogenic products more than three times per day, sweets more than once per week, and higher rates of soda pop increase the severity of dental caries in children (14).

#### Ministerial Regulation on Health no. 41 of 2014 concerning guidelines for balanced nutrition

This policy is intended to provide guidelines for daily food consumption and healthy behavior, including for children (15). This policy describes two visuals used to depict balanced nutrition: the balanced nutrition pyramid and my meal plate. The Balanced Nutrition Pyramide (BNP) has four successive layers from the bottom to the top; the layers grow smaller as one goes up the cone. Four layers mean that BNP is based on the principle of four pillars, namely:

a. Consumption of various foods in a balanced proportion and sufficient (not excessive) quantities that are carried out regularly.
b. Physical activity to balance the expenditure and intake of nutrients, which are the main body's energy resource.
c. Getting used to clean living behavior refers to maintaining cleanliness to avoid infectious diseases.
d. Monitoring indicators of the balance of nutrients in the body, known as body mass index (BMI).

These balanced nutrition guidelines are presented as a visual picture of a balanced nutrition pyramid that serves as a guide for daily consumption. Sugar, salt, and fat are found in the highest position on the pyramid, indicating that consumption of these foods should be limited to small quantities. For each food group, the recommended number of servings is written.

Concerning ECC, this Regulation can be a guide to keeping children's BMI at normal status. Research shows that a BMI above or below normal is associated with caries (16). Consumption of various foods, especially fruits and vegetables, can also prevent caries. The guidelines for clean and healthy living in BNP are also important to be part of the ECC prevention measures. Since dental caries is a multifactorial disease, Toothbrushing is a fundamental self-care behavior for maintaining children's oral health (17).

#### Ministerial Regulation on Health no. 28 of 2019 concerning the recommended nutritional adequacy rate for the Indonesian people

The nutritional adequacy rate or recommended dietary allowance (RDA) is a value that indicates the average need for specific nutrients that must be met every day to maintain a healthy life for all people according to age group, gender, level of physical activity, and physiological conditions. RDA is used at the level of consumption, which includes adequate calories, protein, fat, carbohydrates, fiber, water, vitamins, and minerals. The average consumption level is 2,100 kcal daily to achieve optimal health status (18). The RDA is beneficial as a reference for nutritional labels, establishing balanced nutrition guidelines, and developing a quality index of food consumption which is also beneficial to become a guideline for parents. It will encourage parents to understand the composition of nutrients and identify which elements have the potential contributing factors to ECC.

#### Ministerial Regulation on Health no. 29 of 2019 concerning nutritional management in children with disease-related malnutrition

The central government and local governments are responsible for the implementation of countermeasures to nutritional problems for children due to disease in an integrated and sustainable manner. Diseases, as referred to in this Regulation, include diseases that cause children to be at risk of failure to grow, like malnutrition or congenital metabolic disorders (19). Handling cases is carried out by a team of health workers who each have competence in medicine, nutrition, midwifery, and nursing at a Community Health center or hospital and is carried out through diagnosis of causes and appropriate management of nutritional problems. Regarding ECC, this Regulation is a guide to preventing stunting conditions, which have a significant relationship with the incidence of ECC. Children who need nutritional status improvement will get special medical attention from a medical team that also has a potential role in implementing ECC preventive measures.

#### Regulation of the President of the Republic of Indonesia number 83 of 2017 concerning strategic food and nutrition policy

The implementation of strategic food and nutrition policies aims to improve the nutritional status of Indonesians (20). Efforts to improve community nutrition include: (a) promotion of and education about nutrition for the general public, (b) providing nutritional supplementation, (c) addressing health services and nutrition problems, (d) facilitating community empowerment in the field of food and nutrition, (e) providing social security that supports the improvement of food and nutrition and (f) implementing early childhood education programs. Coordination of food and nutrition development includes food and nutrition planning, strengthening of cross-sectoral roles, strengthening of civil registration in improving nutrition, involvement of stakeholders, and monitoring and evaluation. Nutritional status improvement targets selected groups such as pregnant women, babies, children under 5 years, low-income groups, and people with certain health risks. Healthy food promotion for personal consumption is an important part of community involvement to maintain the population's health, including preventing ECC indirectly.

### Current situation challenges

Studies have found a link between a child's malnutrition AL status and ECC. The stunting and underweight rate in Indonesia is 24.4 and 17.0% for children under the age of five mostly come from low-income families; moreover, the prevalence of obesity is found in 3.8 % of children and is more common in children from wealthy families (21). This condition illustrates the incidence of malnutrition in all socioeconomic groups in Indonesia. Studies in Indonesia and other Asian countries showed that the status of dental caries and odontogenic infections in primary teeth was most common in underweight children and children with stunting (22). One study showed that in primary dentition, dental caries was significantly and inversely related to weight-for-age, height-for-age, and BMI-for-age (23). Obese children are more prone to dental caries (24). A sedentary lifestyle in obese children tends to encourage consuming snacks between meals, which can lead to caries (25).

One challenging condition is the Indonesian people's diet pattern, which is high in carbohydrates (5). Carbohydrates are mostly consumed in the form of rice, which has a fairly large Glycaemic Index (GI) content (5). One study showed that foods with a higher GI might increase the risk of dental caries (26). Another challenge is the increasing rate of high sugar food and beverages consumed by children in recent years in Indonesia (27). The high frequency of sugar intake is a risk factor for the onset of caries in early childhood. This situation may be complicated since the children's dietary patterns are strongly influenced by mothers and other primary caregivers, whose consumed, and preferred foods are based on cultural and societal influence.

## Discussion

The food and nutrition policies implemented in Indonesia are expected to impact ECC prevention positively. One important nutrient responsible for the increasing prevalence of ECC is excessive consumption (quantity or frequency) of sugar (sucrose) (28). Thus, it is interesting that Ministerial Regulation on Health No. 30/ 2013 concerning the Inclusion of Information on Sugar, Salt, and Fat for Processed and Fast Foods states: 'The recommended sugar consumption per person per day is 10% of total energy (200 kcal) or the equivalent of 4 tablespoons/person/day or 50 grams/person/day). This recommendation is in line with the result of a systematic review, which showed that the group that consumed sugar <10% of the total energy needed had a lower prevalence of caries than the group that consumed sugar >10% of the total energy needed (29). It shows that Ministerial Regulation on Health No. 30 of 2013 provides a warning for the community that is in line with controlling the incidence of ECC.

Ministerial Regulation on Health No. 41 of 2014 concerning Guidelines for Balanced Nutrition provides recommendations for the number of servings according to adequate caloric intake levels for various age groups. Unfortunately, there is not enough information about the right amount (units in grams) of food (especially sugar) that children should consume on a daily basis. The explanation of the measured weight is very important, especially for a child's mother/caregiver, because the recommended amount of sugar consumed by children every day (in grams) will impact the incidence of ECC (30). Thus, there needs to be a more concrete rule regarding the exact size and frequency of consuming sugar instead of only focusing on portions.

Moreover, the policies implemented in Indonesia are currently limited to only providing information on the sugar content in foods and beverages sold commercially, not directly regulating restrictions on consumption. Many people find it challenging to decrease their sugar intake on a voluntary basis. Restricting the sugar added to food and beverages sold commercially should become the efforts enacted to prevent caries in children (31). Research conducted in Indonesia shows that some beverages, such as energy drinks and carbonated drinks sold in the country, are high in sugar (27). It is such a concern that the rush of attractive advertisements and aggressive sales models for junk food targeting children causes them to be attracted to that type of food and want to consume it.

Ministerial Regulation on Health No. 28 of 2019 concerning the recommended nutritional adequacy rate for Indonesians must be presented in a simple and easy-to-understand way to meet the children's daily nutritional adequacy rate. It will be very useful for mothers or caregivers and help them identify the right foods and beverages that are good for their children. It is not wise to just let parents focus on how to meet the nutritional needs of children by only informing them about the standards of nutritional adequacy while ignoring the skills they need to choose enjoyable and healthy foods. Combining carbohydrate intake with the consumption of vegetables and fruits is crucial since studies showed that increasing the intake of fibrous foods can produce saliva, which has protective properties against ECC (32).

The Presidential Regulation of the Republic of Indonesia Number 83 of 2017 concerning Strategic Food and Nutrition Policy and Ministerial Regulation on Health No. 29 of 2019 concerning the management of nutrition problems for children due to disease may become the ultimate Regulation on strengthening community empowerment in the field of food and nutrition for having a significant role in preventing ECC. Since health promotion programs and early childhood education have become important strategies, health workers must actively provide consultation on nutrition and good feeding practices for children in the community to prevent ECC. This kind of intervention has significantly reduced the incidence and severity of caries in 4-year-olds in low-income communities (23). Another effort could be to implement a policy of providing healthy food every day in preschools, which can be a model for introducing good food to children and successfully lowering the caries status of children, like in a study in Auckland (33).

It is concluded that since national health programs vary widely in mission, policies related to ECC are not specifically mentioned or discussed in those documents. However, some potential contents of the existing regulations may encourage efforts to prevent ECC.

## Actionable recommendations

1. A more detailed explanation regarding sugar intake restrictions for children in Indonesia is needed. For instance, in the Regulation concerning Guidelines for Balanced Nutrition, there is no detailed explanation on how much the right amount of sugar for a group of children (units in grams) in 1 day. The content contained in the Regulation only describes the size of the number of servings. It is important to set rules for limiting sugar intake based on age to become a guide for parents when choosing safe and healthy foods for children and, further, may control the incidence of Early Childhood Caries (ECC).

2. Guidelines for child feeding practice need to be emphasized more on information related to nutritional adequacy rates in each age group. A guideline is needed for parents to implement good eating patterns for their children, which can avoid the risk of Early Childhood Caries (ECC).

3. Includes Healthy Diet education to prevent ECC, particularly for pre-schoolers' parents/caregivers. Thus, it is hoped that parents or caregivers have more skills in implementing healthy eating parenting patterns for children. Parents also can teach their children to choose healthy snacks and have an impact on reducing the prevalence of ECC.

4. Since high sugar consumption and obesity is the crucial risk factor for ECC, there must be strict supervision and sanctions for the company that sells food and beverage exceeding the maximum standard amounts of sugar, salt, and fat. In addition, there should be coordination in the trade sector, food system, and agricultural policy to protect children's health.

5. Professionals Training for dental health workers to improve skills in providing nutrition education in a dental clinic setting or the community is needed. Dental health workers should provide accurate information about nutrition's influence on ECC occurrence and information related to current nutritional regulations. However, multisectoral approaches that includes health, nutrition and education are needed to address ECC.

It is hoped that the above recommendation efforts to implement policies related to food and nutrition in Indonesia will complement efforts to improve children's dental health, namely to reduce the prevalence of ECC.

## Author contributions

RA came up with the initial concept for the manuscript, produced the preliminary draft, and revised it. According to their areas of competence, FS, MA, and LA added materials. All authors gave the manuscript a close reading to assess its intellectual quality. All authors contributed to the article and approved the submitted version.

## Conflict of interest

The authors declare that the research was conducted in the absence of any commercial or financial relationships that could be construed as a potential conflict of interest.

## Publisher's note

All claims expressed in this article are solely those of the authors and do not necessarily represent those of their affiliated organizations, or those of the publisher, the editors and the reviewers. Any product that may be evaluated in this article, or claim that may be made by its manufacturer, is not guaranteed or endorsed by the publisher.
